# Impact of ABO blood group on the prognosis of patients undergoing surgery for esophageal cancer

**DOI:** 10.1186/s12893-015-0094-1

**Published:** 2015-09-29

**Authors:** Wei Wang, Lei Liu, Zhiwei Wang, Min Wei, Qi He, Tianlong Ling, Ziang Cao, Yixin Zhang, Qiang Wang, Minxin Shi

**Affiliations:** Department of Surgery, The Affiliated Tumor Hospital of Nantong University, Nantong, Jiangsu Province China; Department of Breast, International Peace Maternity and Child Health Hospital, Shanghai Jiao Tong University, Shanghai, China; Department of Thoracic Surgery, Shanghai Renji Hospital Affiliated to Shanghai Jiao Tong University School of Medicine, Shanghai, China

**Keywords:** Esophageal cancer, ABO blood group, Prognosis

## Abstract

**Background:**

ABO blood type is an established prognostic factor in several malignancies, but its role in esophageal cancer (EC) is largely unknown. The aim of this study is to determine whether ABO blood group is associated with survival after esophagectomy for EC.

**Methods:**

A total of 406 patients who underwent surgery for EC were enrolled. The associations of ABO blood group with clinical and pathological variables were assessed using chi-square test. Associations of ABO blood group with the survival were estimated using univariable and multivariable Cox proportional hazards regression models.

**Results:**

The ABO blood group proportionally associated with the grade of EC tumor (*P* = 0.049). The ABO blood group status did not correlate with disease-free survival (DFS) in univariable analysis or multivariable analysis (*P* > 0.05). And there was no significant relationship between the ABO blood group and overall survival (OS) in univariable analysis or multivariable analysis (*P* > 0.05).

**Conclusions:**

Our results suggested that no association between ABO blood group and the survival was observed in patients undergoing surgery for EC.

**Electronic supplementary material:**

The online version of this article (doi:10.1186/s12893-015-0094-1) contains supplementary material, which is available to authorized users.

## Background

Esophageal cancer (EC) was ranked as the eighth most common cancer worldwide, with 482,300 new cases estimated in 2008, and the sixth most common cause of death from cancer with 406,800 deaths [1]. At present, surgery is still the mainstay of treatment for patients with EC. Despite that the surgical techniques have been improved over the past decades, the prognosis of this disease remains poor. One of the reasons is that many cases are at the advanced stage on diagnosis. It is well known that cancer can be caused by the interaction between environmental factors and genetic variations. Up to now, several risk factors related to EC have been previously evaluated, including cigarette smoking, alcohol consumption, low vegetable intake and family history of cancer, BMI and ABO blood group [2–6].

The ABO blood group system was one of the most widely used blood types in clinical practice, which has been discovered over a century. During the past years, several studies have investigated the possible relationship between ABO blood group and the risk of cancer. Individuals with blood group A with an increased incidence were observed in gastric cancer, hepatocellular cancer, pancreatic cancer, ovary cancer and nasopharyngeal cancer [7–11]. These findings indeed reminded us that ABO blood group played an important role in the development of the various human cancers. Therefore, the hypothesis that ABO blood group may also be seen as a candidate prognostic factor of these diseases comes to us. However, no significant association was found between ABO blood group and the survival of gastric cancer or pancreatic cancer [12, 13]. To date, little information about whether the ABO blood group is associated with the survival of EC patients can be obtained.

As a result, the aim of this study was to determine whether ABO blood group system has an effect on clinicopathologic characteristics and prognosis of EC patients.

## Methods

### Patient selection

During the period of patient enrollment, among of 429 cases with symptom, 397 cases were diagnosed as EC, and among of 647 cases without symptom, 24 cases were diagnosed as EC. Fifteen EC cases with symptom were excluded from our study because of the following reasons, received chemotherapy and/or radiotherapy before surgery, with more than one primary cancer, with R1 or R2 resection. Finally, in this retrospective cohort study, we retrieved a total of 406 patients who have undergone esophagectomy for EC at Nantong tumor hospital (between January 2007 and July 2008) and Renji hospital, Shanghai ( between January 2006 and September 2008). The cohort consisted of 275 males and 131 females with the median age of 60 years old (from 25 to 86 years old). EC was confirmed by postoperative histologic pathology in all cases. Tumor stage was classified by the routine histopathologic assessment according to the 7^th^ edition of UICC TNM staging system [14], including 175, 124 and 107 patients with stage I, II, III, respectively. This study was approved by the institutional review board and ethics committee at Nantong tumor hospital (Institutional Review Board of Nantong Cancer Center) and Renji hospital (Specialty Committee on Ethics of Biomedicine Research, Renji, Shanghai). The written informed consents were obtained from all the patients.

### Treatment and information collection

Preoperative evaluation was performed before the decision for surgery. These preoperative risk assessments included a complete medical history and physical examination, complete blood count and serum biochemistry tests, arterial blood gas analysis, ABO and Rh blood group, x-ray, electrocardiogram (ECG), pulmonary function tests, and computed tomography scans of the thorax and the upper abdomen.

For tumors of the upper-third esophagus, the cervico-thoraco-abdominal (right thoracotomy) procedure was performed. For lesions in the mid and lower third, esophagectomy was carried out by the left thoracotomy. Two or three-fielded lymph nodes dissection was performed for each patient. One hundred and twenty-two patients received adjuvant chemotherapy and eighty-four patients received adjuvant radiotherapy after surgery. And the most common chemotherapy regimen consists of 5-FU plus cisplatin for a mean of 3 cycles after surgery, depending on clinical response or the occurrence of adverse effect.

Clinical information was obtained from the medical records. Clinicopathologic features evaluated for each case included the diagnosed age, sex, ABO and Rh blood group status, tumor size, tumor location, clinical stage, tumor grade, histological type, margin status and perioperative blood transfusion.

### Follow up

All the patients remained alive at least 30 days after the surgery, and were followed-up using a standard protocol after discharge from the hospital. The patients received follow up examinations every 3 months for the first 2 years after the operation, every 6 months for the following 3 years, and yearly examinations thereafter. Recording of medical history, physical examination, and CT of the chest were performed during the follow-up time. Endoscopic and whole-body examination was obtained in cases of recurrence or metastasis.

The endpoints were disease-free survival (DFS) and overall survival (OS). DFS was defined as the interval from the date of surgery to the date of local or regional disease recurrence, distant metastasis, or to the last follow-up date. OS was calculated from the time of surgery to the time of death from any cause, or to the time of last follow-up, at which point the data were censored.

The follow-up was performed until the end of September 2013. The median follow-up time was 29 months (range 2 to 92 months). In this cohort, the follow-up information was obtained for 390 cases (96.06 %). And the characteristic of the patients with loss to follow up was displayed in Additional file 1: Table S1. In the current study, there were 251 deaths regardless of the causes. Only 2 of them were due to causes not related to the EC. There were 287 patients who had recurrence or metastasis during the follow-up.

### Statistical analysis

Categorical data were presented as counts and group comparisons were made with the chi-squared test or the Fisher’s exact test. The Kaplan-Meier method was used to construct OS and DFS curves, and the two-side log-rank test was used to determine the statistical significance of differences. The prognostic significance of clinical and pathologic characteristics was determined using univariate Cox regression analysis. Only the factors with significant association in the univariate analysis (*P* < 0.20) and ABO blood group status were included in the multivariate analysis. The outcomes of Cox regression analysis was measured by hazard ratio (HR) and its 95 % confidence intervals (CI). All data were processed using SPSS 15.0 software package. The P values less than 0.05 were considered as significant.

## Results

### ABO blood group and clinicopathologic characteristics

Clinicopathologic characteristics of all subjects stratified by ABO blood group were displayed in Table 1. Among the 406 subjects, 152 (37.4 %) were blood group A, 113 (27.8 %) were blood group B, 114 (28.1 %) were blood group O, and the remaining 27 (6.7 %) were blood group AB. The proportion of poorly-differentiated EC among patients with blood group AB was significantly lower than those with other blood groups (*P* = 0.049). However, no significant difference was observed with regard to age (*P* = 0.669), sex (*P* = 0.511), tumor location (*P* = 0.174), tumor size (*P* = 0.218), T stage (*P* = 0.276), N stage (*P* = 0.924), TNM stage (*P* = 0.367), histopathological type (*P* = 0.218), postoperative adjuvant treatments (*P* = 0.839), vascular invasion (*P* = 0.344) or perioperative blood transfusion (*P* = 0.238).

**Table 1:** Associations of ABO blood group with clinical and pathological variables in 406 patients with EC

Variables	A (%)	B (%)	O (%)	AB (%)	Total (%)	*P*-value
Age (years)	59.2 ± 5.4	60.6 ± 7.5	60.3 ± 7.1	60.1 ± 7.0		0.669
Gender						0.511
Male	105(69.1 %)	66(58.4 %)	84(73.7 %)	20(74.1 %)	275(67.7 %)	
Female	47(30.9 %)	47(41.6 %)	30(26.3 %)	7(25.9 %)	131(32.3 %)	
Location of tumor						0.174
Upper	5(3.3’%)	10(8.8 %)	4(3.5 %)	2(7.4 %)	21(5.2 %)	
Middle	111(73.0 %)	85(75.2 %)	80(70.2 %)	17(63.0 %)	293(72.2 %)	
Lower	36(23.7 %)	18(15.9 %)	30(26.3 %)	8(29.6 %)	92(22.7 %)	
Tumor size						0.218
<5 cm	79(52.0 %)	52(46.0 %)	66(57.9 %)	17(63.0 %)	214(52.7 %)	
≥5 cm	73(48.0 %)	61(54.0 %)	48(42.1 %)	10(37.0 %)	192(47.3 %)	
T stage						0.276
pT1	33(21.7 %)	19(16.8 %)	20(17.5 %)	9(33.3 %)	81(20.0 %)	
pT2	46(30.3 %)	38(33.6 %)	30(26.3 %)	8(29.6 %)	122(30.0 %)	
pT3	67(44.1 %)	44(38.9 %)	54(47.4 %)	8(29.6 %)	173(42.6 %)	
pT4	6(3.9 %)	12(10.6 %)	10(8.8 %)	2(7.4 %)	30(7.4 %)	
N stage						0.924
N0	107(70.4 %)	78(69.0 %)	78(68.4 %)	19(70.4 %)	282(69.5 %)	
N1	27(17.8 %)	19(16.8 %)	21(18.4 %)	5(18.5 %)	72(17.7 %)	
N2	12(7.9 %)	8(7.1 %)	12(10.5 %)	2(7.4 %)	34(8.4 %)	
N3	6(3.9 %)	8(7.1 %)	3(2.6 %)	1(3.5 %)	18(4.4 %)	
TNM stage						0.367
I	71(48.7 %)	45(39.8 %)	46(40.4 %)	13(48.1 %)	175(43.1 %)	
II	44(28.9 %)	42(37.2 %)	30(26.3 %)	8(29.6 %)	124(30.5 %)	
III	37(24.3 %)	36(23.0 %)	38(33.3 %)	6(22.2 %)	107(26.4 %)	
Grade						0.049
Well-differentiated	28(18.4 %)	16(14.2 %)	17(14.9 %)	6(22.2 %)	67(16.5 %)	
Moderately-differentiated	20(58.4 %)	69(61.1 %)	52(45.6 %)	17(63.0 %)	208(51.2 %)	
Poorly-differentiated	54(35.5 %)	28(24.8 %)	45(39.5 %)	4(14.8 %)	131(32.3 %)	
Histopathological type						0.170
Squamous cell carcinoma	140(92.1 %)	105(92.9 %)	100(87.7 %)	27(100.0 %)	372(91.6 %)	
Others^※^	12(7.9 %)	8(7.1 %)	14(12.3 %)	0(0 %)	34(8.4 %)	
Adjuvant treatment						0.839
Yes	54(35.5 %)	40(35.4 %)	44(38.6 %)	8(29.6 %)	146(36.0 %)	
No	98(64.5 %)	73(64.6 %)	70(61.4 %)	19(70.4 %)	260(64.0 %)	
Vascular invasion						
Positive	14(9.2 %)	7(6.2 %)	14(12.3 %)	4(14.8 %)	39(9.6 %)	0.344
Negative	138(90.8 %)	106(93.8 %)	100(87.7 %)	23(85.2 %)	367(90.4 %)	
Blood transfusion						0.212
Yes	8(5.2 %)	6(5.2 %)	12(10.5 %)	2(7.1 %)	32(7.9 %)	
No	144(94.8 %)	107(94.8 %)	102(89.5 %)	25(92.9 %)	374(92.1 %)	

^※^Others included adenocarcinoma, adenosqumaous carcinoma and mucoepdermoid carcinoma

### ABO blood group and disease-free survival

**Fig. 1:**
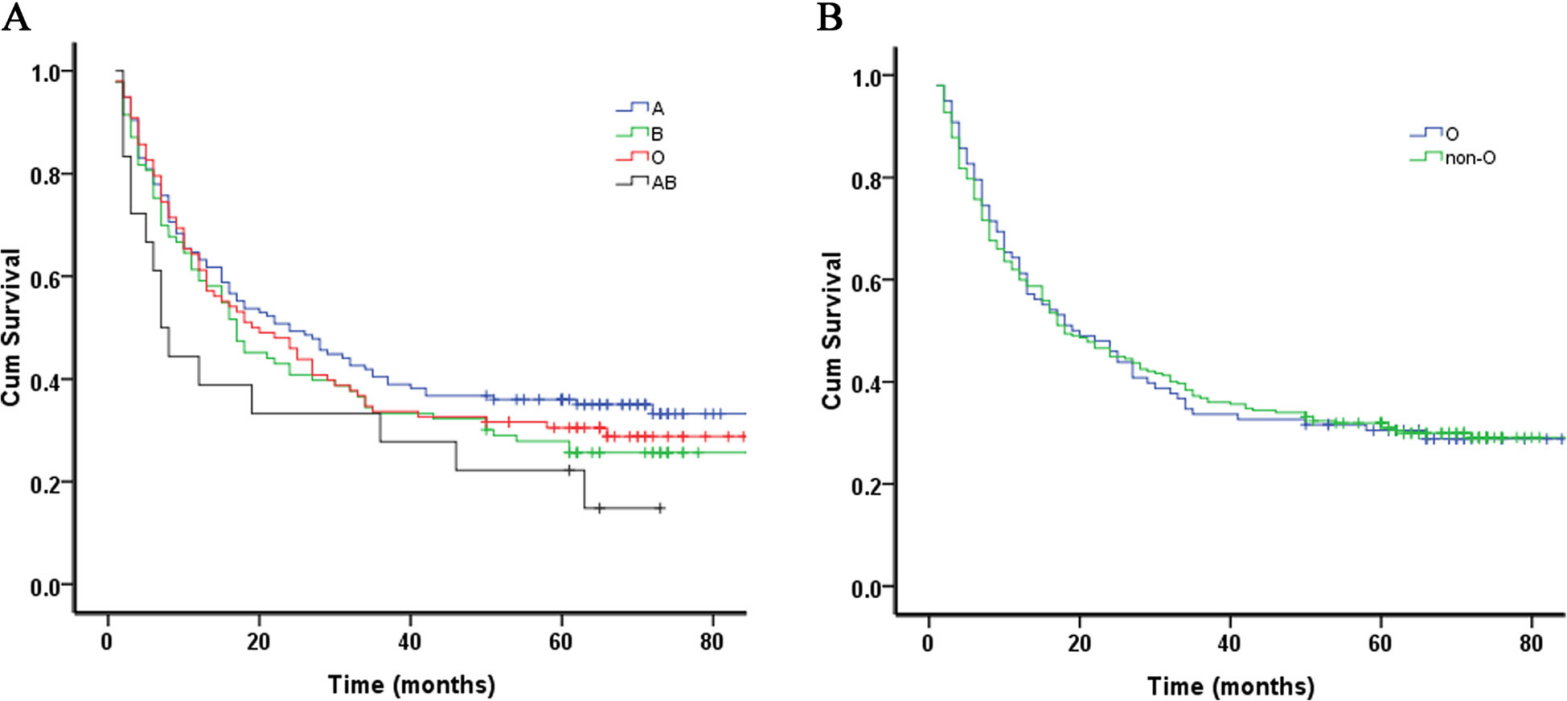
Disease-free survival in 390 patients with esophageal cancer according to ABO blood group. **a** Survival rates of patients with each blood group. **b** Survival rates of patients with blood group O compared with patients with non-O (A, B, and AB) blood groups

A total of 287 patients had recurrence or distant metastasis before the last follow-up. The 5-year DFS rate for EC patients with blood groups A, B, O and AB was 38.8 %, 31.4 %, 32.8 % and 23.8 %, respectively (Table 2). The Kaplan-Meier curves for DFS among different ABO blood group were presented in Fig. 1a. And no significant difference was observed between ABO blood groups and DFS rate for the EC patients (*P* = 0.121). In addition, we divided the whole cases into two subgroups, blood group O in one group, and blood group A, B and AB in the other group. However, there was still no significant difference. And the 5-year DFS rates was 32.8 % and 33.2 % for blood group O and non-O, respectively (*P* = 0.812) (Fig. 1b) (Table 2).

**Table 2:** Distribution of patients and 5-year survival rates by blood group

Blood group	n (%)	5-year DFS (%)	5-year OS (%)
A	146 (37.4)	38.1	46.9
B	108 (27.7)	31.4	38.6
O	109 (27.9)	32.8	42.9
AB	27 (6.9)	23.8	30.3
Non-O	281 (72.1)	33.2	43.2

The relationship between clinicopathologic factors and DFS was assessed by univariate analyses (Table 3). The factors including depth of tumor infiltration, lymph nodes status, TNM stage, tumor grading, adjuvant treatment, vascular invasion, perioperative blood transfusion were significantly correlated with DFS (*P* < 0.05). However, the factor ABO blood group was not significantly associated with DFS (*P* = 0.121). Then the multivariate analysis containing the correlated factors with significant difference and ABO blood group was performed. The result showed that ABO blood group was not a significant prognostic factor for DFS (*P* = 0.215) (Table 3).

**Table 3:** Univariate and multivariate Cox proportional hazards regression for disease-free survival

Variables	Category	Univariate analysis			Multivariate analysis		
		HR	95 % CI	*P-*value	HR	95 % CI	*P-*value
T stage	pT1+ pT2	1.00			1.00		
	pT3+ pT4	2.24	1.72–2.90	<0.001	1.79	1.21–2.64	0.004
N stage	N0	1.00			1.00		
	N1-3	2.38	1.83–3.09	<0.001	1.66	0.82–3.34	0.156
TNM stage	I + II	1.00			1.00		
	III	2.67	2.04–3.49	<0.001	1.72	1.17–2.53	0.006
Grade	G1	0.73	0.49–1.08		0.80	0.54–1.20	
	G2	1.00			1.00		
	G3	1.44	1.10–1.88	0.001	1.32	0.95–1.71	0.121
Vascular invasion	Negative	1.00			1.00		
	Positive	1.50	1.01–2.22	0.045	1.47	0.79–2.76	0.221
Adjuvant treatment	No	1.00			1.00		
	Yes	2.65	1.83–3.82	<0.001	2.19	1.49–3.22	<0.001
Blood transfusion	No	1.00			1.00		
	Yes	1.92	1.03–3.57	0.037	2.33	1.21–4.51	0.012
ABO blood group	A	1.00			1.00		
	B	0.97	0.71–1.34		0.94	0.60–1.47	
	O	1.07	0.79–1.47		1.06	0.67–1.68	
	AB	1.78	1.08–2.99	0.121	2.10	1.00–4.41	0.215
ABO blood group	O	1.00			-		
	A/B/AB	1.04	0.78–1.37	0.812	-		

### ABO blood group and overall survival

**Fig. 2:**
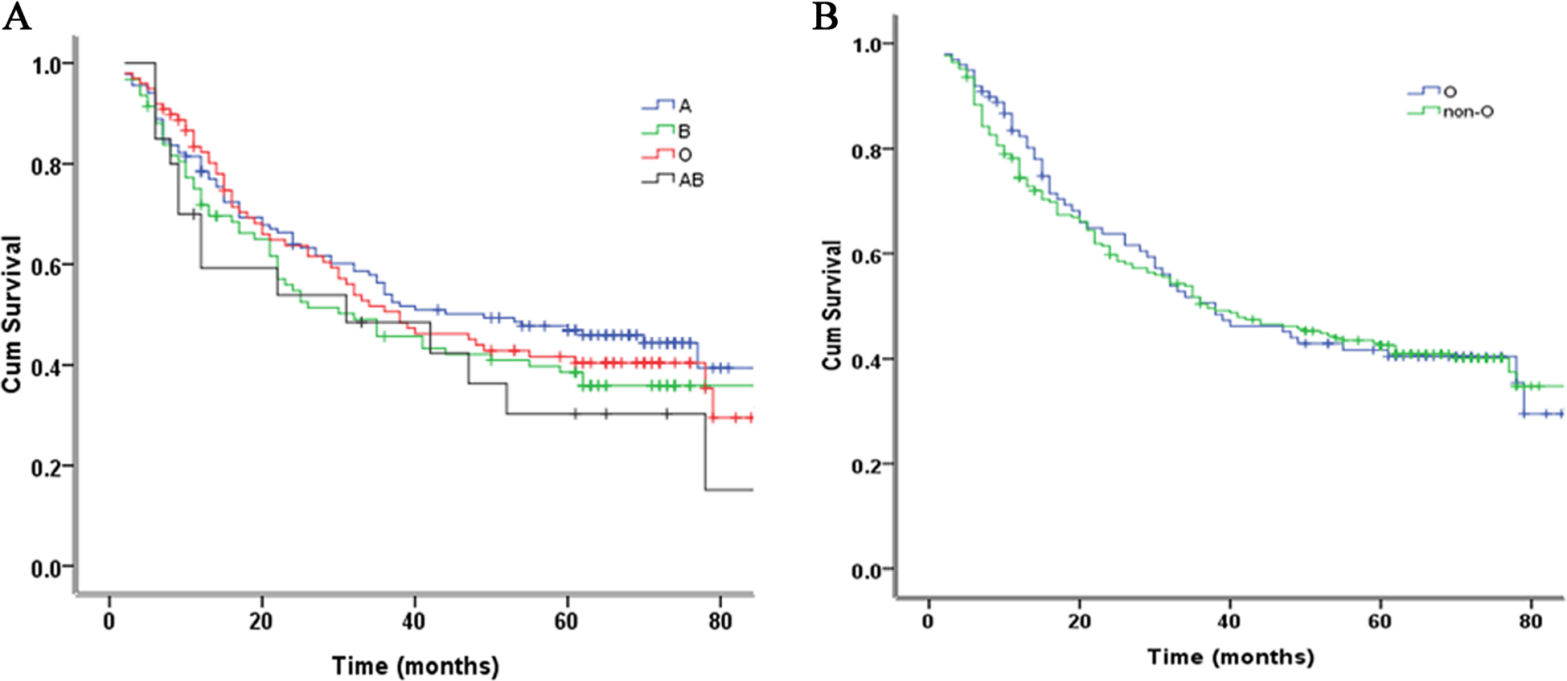
Overall survival in 390 patients with esophageal cancer according to ABO blood group. **a** Survival rates of patients with each blood group. **b** Survival rates of patients with blood group O compared with patients with non-O (A, B, and AB) blood groups

The median follow-up period for the entire study population was 29 months (range, 2-92 months). Death disregarding the causes occurred in 251 of 402 enrolled patients at the time of the final analysis. There were only two patients who died due to causes not related to cancer (one from suicide and the other from cerebral hemorrhage). The 5-year OS rate for blood group A, B, O, and AB was 46.9 %, 38.6 %, 42.9 % and 30.3 %, respectively (Table 2). As reflected in Fig. 2a, there was no significant difference in survival among the diversified blood groups (*P* = 0.254). Moreover, we compared the OS for blood group O with non-O groups (A, B and AB). And the 5-year OS rate for blood group O and non-O was 42.9 % and 43.2 %, respectively. Significant difference was still not found between the groups (*P* = 0.846) (Fig. 2b) (Table 2).

The univarite analysis suggested that the factors of T stage, lymph nodes metastasis, TNM stage, grade, postoperative adjuvant treatment, vascular invasion, perioperative blood transfusion were strong predictors of long-term OS (*P* < 0.20), whereas the factor ABO blood group was not significantly correlated with the OS (*P* = 0.254) (Table 4). The multivariate analyses contained those factors with significant difference in univarite analysis and ABO blood group. The multivariate analyses showed that ABO blood group was not significantly associated with OS (*P* = 0.065) (Table 4).

**Table 4:** Univariate and multivariate Cox proportional hazards regression for overall survival

Variables	Category	Univariate analysis			Multivariate analysis		
		HR	95 % CI	*P-*value	HR	95 % CI	*P-*value
T stage	pT1+ pT2	1.00			1.00		
	pT3+ pT4	2.64	1.91–3.65	<0.001	1.82	1.23–2.70	0.002
N stage	N0	1.00			1.00		
	N1-3	2.35	1.71–3.23	<0.001	1.62	0.80–3.29	0.161
TNM stage	I + II	1.00			1.00		
	III	2.61	1.88–3.61	<0.001	1.65	1.13–2.42	0.011
Grade	G1	0.75	0.50–1.05		0.82	0.56–1.22	
	G2	1.00			1.00		
	G3	1.37	1.02–1.81	0.058	1.31	0.96–1.70	0.134
Vascular invasion	Negative	1.00			1.00		
	Positive	2.71	1.66–3.86	<0.001	1.47	0.79–2.76	0.221
Adjuvant treatment	No	1.00			1.00		
	Yes	2.76	2.02–3.78	<0.001	2.30	1.67–3.17	<0.001
Blood transfusion	No	1.00			1.00		
	Yes	2.40	1.47–3.94	<0.001	2.51	1.50–3.88	<0.001
ABO blood group	A	1.00			1.00		
	B	1.25	0.89–1.77		1.43	1.01–2.06	
	O	1.10	0.78–1.55		1.14	0.80–1.62	
	AB	1.72	0.95–3.10	0.254	1.96	0.98–3.52	0.065
ABO blood group	O	1.00			-		
	A/B/AB	1.03	0.76–1.40	0.846	-		

### Sensitivity analysis

To evaluate the stability of our findings, sensitivity analysis was carried out by excluding those 24 EC cases without symptom. Among the 382 cases, 143 (37.4 %) were blood group A, 106 (27.7 %) were blood group B, 108 (28.3 %) were blood group O, and the remaining 25 (6.5 %) were blood group AB. With regard to disease-free survival, neither the univariate analyses nor the multivariate analyses showed that ABO blood group was a significant prognostic factor (*P* = 0.138 and *P* = 0.241). As for overall survival, neither the univariate analyses nor the multivariate analyses showed that ABO blood group was significantly associated with OS (*P* = 0.276 and *P* = 0.068). The results were not materially altered, indicating the robust stability of the current findings.

## Discussion

ABO blood group has played an important role in transfusion medicine, which is widely used during clinical practice. Recently, much attention has been given to the connection between ABO blood group and the prognosis of cancer. Previously published studies have confirmed that ABO blood group was significantly associated with the prognosis and could be considered as one of the predictive factors in pancreatic cancer, bladder cancer and renal cell cancer [15–18]. However, there was little information regarding the relationship between ABO blood group and the outcomes of patients with EC.

To our best knowledge, it is the first for us to assess the possible association between ABO blood group and the DFS and OS for EC in China. In the current study, we retrospectively analyzed the data from 406 patients undergoing surgical therapy for EC in two centers. Except for the grade of tumor, we found no statistically significant association with clinical or pathological parameters. We did not observe significant associations between ABO blood type and DFS of EC. And no significant difference of OS was detected among different ABO blood groups in univariate analyses. However, in the multivariate analyses, our findings showed that patients with blood group B or AB had a worse OS compared with those with blood group A. The reasons why this difference in survival is not evident in univariate analyses remain unclear at this stage. Our findings were not consistent with a few previously published studies. In a sample of 496 patients who underwent esophagectomy, Yang and his colleagues found that patients with blood group O had a significantly worse overall survival than non-O blood groups [19]. However, the findings obtained in our study agreed with the results from other malignances, such as breast cancer and lung cancer [20–22], which also suggested that ABO blood group had no significant effect on the outcomes. It could be seen that the outcomes among diversified studies were indeed conflicting. The discrepancies might be due to various genetic backgrounds, retrospective data collection and inconsistent evaluation of endpoints. Due to only one patient with negative Rh blood type among the study population, any correlation of Rh blood group with biological behaviors of EC could not be evaluated in our study.

Direct biologic mechanisms underlying the association between ABO blood group and cancer are inconclusive. However, there are several hypotheses which may explain the relationships observed. The ABO gene is located on the Chromosome 9q and consists of 7 exons. It encodes a glycosyltranferase catalyzing the transfer of carbohydrates to the H antigen, thus forming the antigenic structure of the ABO blood groups [23–25]. Blood group antigens are expressed not only on the surface of red blood cells, but also on numerous other tissues, including esophageal epithelium [26]. Notably, previous report indicated that the loss of blood group H antigen occurred during carcinogenesis of the esophageal mucosa [27]. It has been shown that the modified expression of blood group antigens on the surface of tumor cells may alter cell motility, resistance to apoptosis and immune escape [28]. Additionally, recent studies have revealed the relationship between ABO group genotype and circulating levels of soluble intercellular adhesion molecule-1 (sICAM-1) [29, 30]. Increased levels of sICAM-1 are known to be correlated with a number of human malignancies and may play a role in escape from immune surveillance by tumor cells [31].

Several limitations should be noted in the current study. Firstly, the sample size of the entire cohort was relatively small. There were only 390 patients with complete information allowing for the multivariable analysis, although a majority of the patients in our study had follow-up information available. Secondly, similar to any retrospective study, there was the possibility of selection bias. Those uninvolved population during the study period were noted to have a worse outcome. There was no chance for those who with distant metastasis to have a radical operation, thus resulting in selection bias. Thus, prospective studies focused on this topic should be further investigated. Thirdly, some cases without symptom may be omitted from our study, which biases our results. During the period of cases enrollment, those who were diagnosed as precancerous lesion for EC were followed up. Finally, a few of such cases were also included in the study, because of developing into EC. To some extent, this could decrease the rate of missed diagnosis. Last but not least, it is difficult for us to control the various adjuvant treatment regimens administered, although the chemotherapy regiment for most patients was 5-FU plus cisplatin. Hence, this needs to be kept in mind when interpreting our results.

## Conclusion

In summary, our results failed to suggest an association between ABO blood group and disease-free or overall survival in patients with EC.

## Additional file

Additional file 1: Table S1. The clinical and pathological variables in follow-up and loss to follow-up patients with EC. (DOC 53 kb)
