# Supplementary material for: Impact of ABO blood group on the prognosis of patients undergoing surgery for esophageal cancer
Source: BMC Surg. 2015 Sep 29;15:106. doi: 10.1186/s12893-015-0094-1 (PMC4588230; doi:10.1186/s12893-015-0094-1)
Supplement: Additional file 1: Table S1. — The clinical and pathological variables in follow-up and loss to follow-up patients with EC. (DOC 53 kb) [file 12893_2015_94_MOESM1_ESM.doc]

Additional file 1: Table S1. The clinical and pathological variables in follow-up and loss to follow-up patients with EC

| Variables | Follow-up group (n=390)(%) | Loss to follow-up  group (n=16)(%) | P-value |
| --- | --- | --- | --- |
| Age (years) | 60.2±7.1 | 60.5±7.5 | 0.869 |
| Gender |  |  | 0.664 |
| Male | 264(67.7%) | 10(62.5%) |  |
| Female | 126(32.3%) | 6(37.5%) |  |
| Location of tumor |  |  | 0.719 |
| Upper | 20(5.1%) | 1(6.3%) |  |
| Middle | 282(72.3%) | 11(68.8%) |  |
| Lower | 88(22.6%) | 4(25.0%) |  |
| Tumor size |  |  | 0.825 |
| ＜5cm | 206(52.8%) | 8(50.0%) |  |
| ≥5cm | 184(47.2%) | 8(50.0%) |  |
| TNM stage |  |  | 0.367 |
| I | 168(43.1%) | 7(43.8%) |  |
| II | 119(30.5%) | 5(31.3%) |  |
| III | 103(26.4%) | 4(25.0%) |  |
| Grade |  |  | 0.049 |
| Well-differentiated | 64(16.4%) | 3(18.8%) |  |
| Moderately-differentiated | 201(51.5%) | 7(43.8%) |  |
| Poorly-differentiated | 125(32.1%) | 6(37.5%) |  |
| Histopathological type |  |  | 0.170 |
| Squamous cell carcinoma | 357(91.5%) | 15(93.8%) |  |
| Others^※^ | 33(8.5%) | 1(6.3%) |  |
| Adjuvant treatment |  |  | 0.839 |
| Yes | 138(35.4%) | 8(50.0%) |  |
| No | 252(64.6%) | 8(50.0%) |  |
| Vascular invasion |  |  |  |
| Positive | 37(9.5%) | 2(12.5%) | 0.344 |
| Negative | 353(90.5%) | 14(87.5%) |  |
| Blood transfusion |  |  | 0.238 |
| Yes | 31(7.9%) | 1 (6.3%) |  |
| No | 359(92.1%) | 15(93.8%) |  |
| ABO blood group |  |  | 0.922 |
| A | 146(37.4%) | 6(37.5%) |  |
| B | 108(27.7%) | 5(31.3%) |  |
| O | 109(27.8%) | 5(31.3%) |  |
| AB | 27(6.9%) | 0(0.0%) |  |

- Others included adenocarcinoma, adenosqumaous carcinoma and mucoepdermoid carcinoma.
